# POAS4SPM: A Toolbox for SPM to Denoise Diffusion MRI Data

**DOI:** 10.1007/s12021-014-9228-3

**Published:** 2014-07-05

**Authors:** Karsten Tabelow, Siawoosh Mohammadi, Nikolaus Weiskopf, Jörg Polzehl

**Affiliations:** 1 WIAS Berlin, Mohrenstr. 39, 10117 Berlin, Germany; 2 Wellcome Trust Centre for Neuroimaging, UCL Institute of Neurology, 12 Queen Square, London, WC1N 3BG UK

**Keywords:** dMRI, Noise reduction, POAS, msPOAS, SPM (RRID:nif-0000-00343)

## Abstract

We present an implementation of a recently developed noise reduction algorithm for dMRI data, called multi-shell position orientation adaptive smoothing (msPOAS), as a toolbox for SPM. The method intrinsically adapts to the structures of different size and shape in dMRI and hence avoids blurring typically observed in non-adaptive smoothing. We give examples for the usage of the toolbox and explain the determination of experiment-dependent parameters for an optimal performance of msPOAS.

## Introduction

Diffusion-weighted magnetic resonance imaging (dMRI) has developed into an extremely versatile tool for the in-vivo structural analysis of tissue, for example in the human brain (Johansen-Berg and Behrens 2009). One reason is that the diffusion signal obtained with the pulsed gradient spin sequence echo (PGSE, Stejskal and Tanner 1965) directly relates, via three-dimensional Fourier transform, to the diffusion propagator which is the probability density of the underlying Random Walk the spin particles experience (Mitra and Sen 1992). Therefore, if we measured the diffusion signal for all possible diffusion gradient directions, times and strengths, i.e. cover the whole *q*-space, we would know the full propagator. Its spatial and directional dependence would allow us to infer on boundaries for the diffusing particles and hence the underlying structure. However, in practice, only a limited coverage of the *q*-space is feasible. Therefore, a number of models have been developed in the past, which reveal at least partial information contained in the diffusion propagator. Most require dMRI data measured on at least one *q*-shell, that is characterized by a single *b*-value (Basser et al. 1994b) subsuming diffusion gradient strength and diffusion time.

The most prominent example of a diffusion model gives rise to Diffusion Tensor Imaging (DTI, Basser et al. 1994a, b). Surprisingly, although this model actually describes free diffusion in anisotropic media it has proven to relate well to the underlying tissue geometry in the brain in general, and to the main fiber directions in the white matter in particular (Johansen-Berg and Behrens 2009). More sophisticated descriptions of the diffusion signal have been examined to infer on more complicated sub-voxel structure like multiple fiber directions. These include HARDI (Frank 2001; Tuch et al. 2002), tensor mixture models (Behrens et al. 2003; Assaf and Basser 2005; Tabelow et al. 2012), higher order tensor approximations (Özarslan and Mareci 2003; Liu et al. 2003; Jensen et al. 2005) and methods to determine the full diffusion propagator (Özarslan et al. 2006; Cheng et al. 2010), see Assemlal et al. (2011) for a recent review.

In any case the measures of interest, like fiber directions or quantitative measures like the fractional anisotropy (FA) in DTI, are estimated based on the raw diffusion images. Hence, the accuracy of the estimates depends on the data quality, which in turn typically requires retrospective correction of artifacts due to eddy-currents, motion (Mohammadi et al. 2010), susceptibility-related distortions (Ruthotto et al. 2012, 2013), instrumental (Mohammadi et al. 2012a, b) or physiological noise (Mohammadi et al. 2013a, b). Signal-to-noise ratio (SNR) is especially low in dMRI because of the additional exponential dependence in the diffusion-weighted signal, see, e.g., Stejskal and Tanner (1965). As a result the SNR in dMRI decreases with increased diffusion weighting, i.e. higher *q*-shells have lower SNR. Furthermore, beyond-tensor models with a larger number of parameters are more sensitive to data noise, making sophisticated denoising strategies mandatory for cutting-edge dMRI. In order to reduce noise in dMRI data a number of different approaches have been developed in recent years starting from Gaussian filtering (Westin et al. 1999), smoothing procedures in tensor space for DTI (Fletcher 2004), to denoising algorithms based on partial differential equations (Ding et al. 2005; Parker et al. 2000; Duits and Franken 2011) to name only a few.

Recently, we developed a position-orientation adaptive smoothing algorithm (POAS, Becker et al. 2012) based on the propagation-separation (PS) approach (Polzehl and Spokoiny 2006; Becker and Mathé 2013). The method is edge-preserving and avoids blurring of the fine anisotropic structures observed in dMRI. The method has been extended to be applicable to dMRI measured on multiple shells and named multi-shell POAS (msPOAS, Becker et al. 2014). It capitalizes on the additional information on the different shells. Furthermore, several improvements make msPOAS feasible from a computational point of view. Finally, msPOAS can also be applied to single-shell dMRI data. Thus, in this paper we will only consider the more general msPOAS approach.

Compared to previous diffusion-model-based adaptive smoothing methods, e.g., Tabelow et al. (2008), (ms)POAS has the advantage that it directly denoises dMRI data without requiring any diffusion-model assumptions. Thus, it avoids a bias towards any diffusion model such as DTI, HARDI, or tensor mixture models (see Becker et al. (2012) for details).

(Ms)POAS has been originally implemented in the **R** language and environment for statistical computing and graphics (R Development Core Team 2014). While the corresponding package **dti** (Tabelow and Polzehl 2014) is easy to install (Polzehl and Tabelow 2011) and allows for applying msPOAS to dMRI data using very few code lines, the use of **R** in the neuroimaging community is still limited. We therefore implemented msPOAS as a toolbox for Statistical Parametric Mapping (SPM) (Friston et al. 2006), the most widely used neuroimaging analysis package, to make the method available to a broader audience, see www.diffusiontools.com.

In this paper we shortly review the method in a simplified way, present the new toolbox for SPM, describe the usage of the toolbox, and suggest methods to determine experiment-dependent measures and to set method parameters. We present some worked examples with single- and multi-shell data. For a more in-depth review of the theory of POAS and msPOAS we refer the reader to the original work in Becker et al. (2012, 2014).

## Methods

MsPOAS is a noise reduction method for dMRI data that is measured on at least one *q*-shell, i.e., for at least one *b*-value and a sufficiently large number of diffusion gradient directions. Although msPOAS also works for very few diffusion gradient directions, it particularly benefits from the information from more gradient directions (Becker et al. 2014). MsPOAS is also suitable for single-shell dMRI data.

### Review: Multi-Shell Position-Orientation Adaptive Smoothing (msPOAS)

The design space in dMRI for a single *q*-shell forms an ${\mathbb{R}}^3\times {\mathbb{S}}^2$ space that contains a collection of points described by their voxel positions in *ℝ* ^3^ and gradient directions in ${\mathbb{S}}^2$ (Duits and Franken 2011). Let *S*_*b*_(*m*) denote the observed signal for the *b*-value and point *m* defined as $m=\left(\mathrm{v}\in {\mathbb{R}}^3,\mathrm{g}\in {\mathbb{S}}^2\right)$, where v is its voxel location and g is its gradient direction. The non-diffusion weighted *b*_0_-image is denoted by *S*_0_(v, 0) = *S*_0_(v) and does of course not depend on g. In case of several *b*_0_-images we consider their mean. Interpolation is used to account for discrepancies in gradient directions across shells, see Becker et al. (2014).

The measured random values *S*_*b*_(*m*) are distributed according to some probability distribution that is parametrized by a “true” intensity parameter *θ*, the noise standard deviation *σ* and a number of degrees of freedom 2*L* where *L* denote the effective number of coils for parallel imaging (Aja-Fernández et al. 2009). Then *S*_*b*_(*m*)/*σ* is assumed to be non-central *χ*-distributed with 2*L* degrees of freedom.

In msPOAS we assume that similar signal values in ${\mathbb{R}}^3\times {\mathbb{S}}^2$ extend over sets of neighboring points *n* = (v_*n*_, g_*n*_). This can be used to obtain an improved estimate for the image value at any point $m\in {\mathbb{R}}^3\times {\mathbb{S}}^2$. If the definition of the neighborhood is specific for the point (and based on the data) we call the method adaptive, or non-adaptive otherwise. The notion of neighborhood typically requires the definition of a metric in the considered design space, here ${\mathbb{R}}^3\times {\mathbb{S}}^2$.

MsPOAS is derived from non-adaptive kernel estimators (Nadaraya 1964; Watson 1964), see also (Fan and Gijbels 1996). Then, for a given distance *δ*(*m*, *n*) between design points *m* and *n*, a non-adaptive kernel estimate ${\overline{S}}_b(m)$ for the expected value at some bandwidth *h* is given by a weighted mean

1 $$ {\overline{S}}_b(m)={\displaystyle \sum_n{\overline{w}}_{mn}{S}_b(n)/{\displaystyle \sum_n{\overline{w}}_{mn}}} $$

with

2 $$ {\overline{w}}_{mn}={K}_{\mathrm{loc}}\left(\frac{\delta \left( m, n\right)}{h}\right) $$

where *K*_loc_ is some kernel function. Instead of the commonly used Gaussian kernel function we employ

$$ {K}_{\mathrm{loc}}(x)={\left(1-{x}^2\right)}_{+} $$

(with (*x*)_+_ denoting the maximum of *x* and 0), due to its higher efficiency and computational simplicity, see Fan and Gijbels (1996).

MsPOAS is based on the propagation-separation approach (Polzehl and Spokoiny 2006; Becker and Mathé 2013) and makes the following important extensions to the non-adaptive estimator:

1. Re-define the weighting schemes given in Eq. (2) by an additional term that evaluates the distance of the signal in two measurement points *m* and *n* making the weights adaptive, see Eq. (4), cf. the construction of bilateral filters.
2. Repeat the estimation step in Eq. (1) using adaptive weights (4) for an increasing (typically geometric) sequence of bandwidths *h*_*k*_ for *k* = 1, …, *k* ^⋆^ instead of a single bandwidth *h*, see Eq. (2), in an iterative procedure, see (Becker et al. 2014). This approach is, therefore, a scale-space method. The specific choice of the sequence of bandwidths is described in detail in Appendix B, Becker et al. (2014).

MsPOAS then calculates at each iteration step *k* new estimates

3 $$ {\tilde{S}}_b^{(k)}(m)={\displaystyle \sum_n{\tilde{w}}_{mn}^{(k)}{S}_b(n)/{\displaystyle \sum_n{\tilde{w}}_{mn}^{(k)}}} $$

with adaptive weighting schemes

4 $$ {\tilde{w}}_{mn}^{(k)}={K}_{\mathrm{loc}}\left(\frac{\delta \left( m, n\right)}{h_k}\right){K}_{\mathrm{ad}}\left(\frac{s_{mn}^{(k)}}{\lambda}\right) $$

employing a second kernel function

$$ {K}_{ad}(x)=\left\{\begin{array}{ll}1\hfill & \mathrm{for}\kern0.5em 0\le x<0.5,\hfill \\ {}{\left(2-2 x\right)}_{+}\hfill & \mathrm{for}\kern0.18em 0.5\ge x.\hfill \end{array}\right. $$

The summation in Eq. (3) is over all signal from the same *q*-shell. For *b*_0_-images averaging in Eq. (3) is reduced to voxel space, see Becker et al. (2014) for details.

In msPOAS we use a simple approximate distance in the design space ${\mathbb{R}}^3\times {\mathbb{S}}^2$:

5 $$ {\delta}_{\kappa}\left( m, n\right)=\left\Vert {\mathrm{v}}_m-{\mathrm{v}}_n\right\Vert +{\kappa}_k^{-1}\mathrm{acos}\left(\left|{\mathrm{g}}_m^T{\mathrm{g}}_n^T\right|\right) $$

where ||. || denotes the *L*_2_-norm in *ℝ* ^3^ and *κ* steers the influence of the geodesic distance on the sphere ${\mathbb{S}}^2$ (Hagmann et al. 2006; Becker et al. 2014). Specifically we use a decreasing sequence *κ*_*k*_ = *κ*_0_/*h*_*k*_ to restrict the smoothing on the sphere at later iteration steps, see Becker et al. (2014).

The *statistical penalty s*_*mn*_^(*k*)^ evaluates the difference between the estimators in points *m* and *n* in the previous iteration step and is defined by

$$ \begin{array}{c}\hfill {s}_{m n}^{(k)}:={\displaystyle \sum_{b\ge 0}{\tilde{N}}_{m, b}^{\left( k-1\right)}\mathcal{KL}\left(\frac{{\tilde{S}}_b^{\left( k-1\right)}(m)}{\sigma},\frac{{\tilde{S}}_b^{\left( k-1\right)}(n)}{\sigma}\right)}\hfill \\ {}\hfill {\tilde{N}}_{m, b}^{(k)}=\underset{k^{\prime}\le k}{ \max }{\displaystyle \sum_n{\tilde{w}}_{m n}^{\left( k\prime \right)}}\hfill \end{array} $$

where $\mathcal{KL}$ denotes the Kullback–Leibler divergence between two non-central *χ*-distribution with 2*L* degrees of freedom and its expectation given as argument. This term has no analytic expression, in msPOAS we use the approximation

6 $$ {s}_{m n}^{(k)}={\displaystyle \sum_{b\ge 0}{\tilde{N}}_{m, b}^{\left( k-1\right)}\frac{2{\displaystyle {\left(\frac{{\tilde{S}}_b^{\left( k-1\right)}(m)}{\sigma}-\frac{{\tilde{S}}_b^{\left( k-1\right)}(n)}{\sigma}\right)}^2}}{{\mathrm{sd}}_L^2\left(\frac{{\tilde{S}}_b^{\left( k-1\right)}(m)}{\sigma}\right)+{\mathrm{sd}}_L^2\left(\frac{{\tilde{S}}_b^{\left( k-1\right)}(n)}{\sigma}\right)},} $$

with *sd*_*L*_(*x*) denoting the standard deviation of a non-central *χ*-distribution with 2*L* degrees of freedom and expectation *x*. The factor ${\tilde{N}}_{m, b}^{\left( k-1\right)}$ characterizes the variance reduction at each shell achieved due to averaging. For all specific details of msPOAS which are not covered here necessary for the handling of the *b*_0_-images, the modification for interpolated signal values and the initialization of the method, we refer to Becker et al. (2014).

### Toolbox Implementation and Installation

The toolbox POAS4SPM for the neuroimaging software SPM has been implemented using C and Matlab. It comes as open-source software with GPL2.

The toolbox is part of the ACID toolbox for “Artefact correction in diffusion MRI” and can be downloaded from its homepage at http://www.diffusiontools.com/. It is listed on the SPM extension homepage http://www.fil.ion.ucl.ac.uk/spm/ext/, too. Installation is done by extracting the toolbox into the toolbox folder of SPM, and compiling all mex-files in the Preprocessing/POAS subfolder. Running the make_ACID.m utility in the cfiles folder of the ACID toolbox will automatically compile all necessary c-files.

### Usage of the Toolbox

As part of the ACID-Toolbox, the msPOAS module runs in the batch editor of SPM. In the menu of the batch editor msPOAS can be found at SPM -> ACID Toolbox -> Pre-Processing -> Choose POAS options -> POAS. One may load/save a batch file to use standard toolbox settings as usual. The toolbox options are defined as follows:

- *Diffusion weighted images*: Choose the *N* images including *N*_*g*_ diffusion weighted and *N*_0_ non-diffusion weighted data files. Data should be given in separate 3D volumes.
- *Diffusion directions*: Add a 3 × *N*–array consisting of the diffusion gradient directions with normalized vectors that appear in the same order as the DTI images were entered. Choose a vector with three zeros for each *b*_0_-image. If a name of a variable is entered here, e.g., with gradient direction data read from a file, its value is automatically evaluated.
- *b-values*: Add a 1 × *N*–array with *b*-values. They should appear in the same order as the DTI images were entered. The *b*-value is given in units of s/mm^2^. *b*_0_-images should have *b* = 0. If the data contains images with a small *b*-value (*b* < 100s/mm^2^), which serve as reference image without directional information, mark them by using *b* = 0 as well. The diffusion-weighted images corresponding to different shells will be identified by their *b*-value. Also here, a variable name can be entered.
- *k star*: This is the parameter *k* ^⋆^ of msPOAS that defines the number of iterations and thus the maximal location bandwidth ${h}_{k^{\star }}$.
- *kappa*: This is the parameter *κ*_0_ of msPOAS that defines the initial ratio of the spatial and spherical distance in Eq. (5).
- *lambda*: This is the adaptation parameter *λ* of msPOAS, see Becker et al. (2012, 2014) for more details.
- *sigma*: The value *σ* is the noise level in the data and must be obtained from the data, see option Estimate sigma in the toolbox. Although the value of *σ* may vary spatially due to effects of parallel imaging, the current implementation of msPOAS assumes a homogeneous variance. The effect of a misspecified *σ* can be partly compensated by the choice of *λ* (Becker et al. 2014).
- *ncoils*: This parameter specifies the parallel imaging factor *L*, i.e., the number of different receiver coils that contributed to the measured signal value. It may also vary with spatial location, but the current implementation of msPOAS assumes a global value for *L*. msPOAS has been shown to be relatively robust against misspecifications of *L* (Becker et al. 2014).

After running the batch script, the smoothed diffusion weighted volumes are written to disk using “poas” as a prefix. Only one *b*0-image, obtained as smoothed average of all original *b*0-images, is written to disk, see Becker et al. (2012). For further processing the corresponding gradient orientations and *b*-values are written to disk as a **.mat**-file. The input directory is used as the target directory for the script’s output.

### Choice of the Method Parameters *k*^⋆^, *κ*_0_, *λ*

**Fig. 1:**
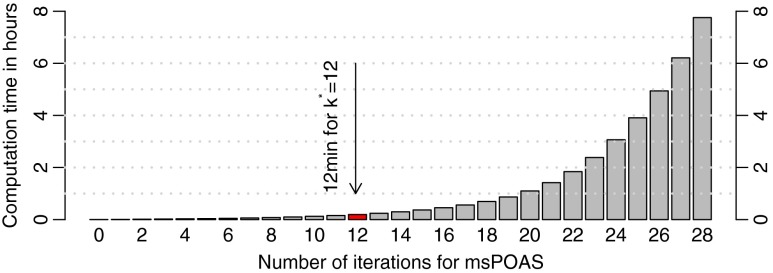
Computation time in hours as a function of the number of iteration steps *k* ^⋆^ for the single-shell dataset used in this paper and a typical set of parameters *κ*_0_ = 0.5, *λ* = 12. The highlighted k^⋆^ = 12 provides a good balance between achieved variance reduction, variance controlled bias and computation time

The number of iteration steps *k* ^⋆^ defines the maximum variance reduction in homogenous image regions, but also the numerical complexity of the method and thus the computation time, see Fig. 1. Large values of *k* ^⋆^ may also lead to a step function approximation with a small step size. We therefore suggest a value between 10 and 12 for *k* ^⋆^.

**Fig. 2:**
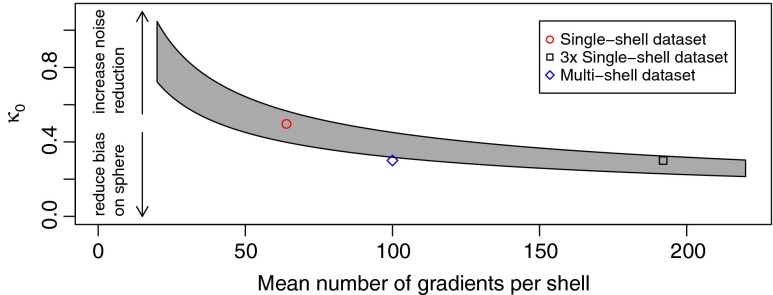
The choice of *κ*_0_ depends on the mean number $\widehat{N}$of diffusion gradient directions per shell and a balance between achieved noise reduction and bias on the sphere. Choices for the datasets in this paper are indicated

The value for *κ*_0_ defines the amount of initial smoothing on the sphere of diffusion weighting directions in the first step *k* = 0. This spherical smoothing stabilizes the initial estimates of msPOAS for a better noise reduction especially at very low SNR. On the other hand spherical smoothing comes with a cost of potential bias and loss of spherical resolution. In successive steps msPOAS therefore restricts the amount of spherical smoothing by the specific choice of the sequence *κ*_*k*_. Due to the decreasing variance of the estimates in the iteration process the statistical penalty in Eq. (6) generally increases. This leads to more restrictive adaptive weights and thus to reduced smoothing on the sphere. We recommend a value for *κ*_0_ such that in the initialization step *k* = 0 for msPOAS an average number of 5 to 10 signal values on the sphere for neighboring gradient directions are included in the smoothing. This is equivalent to a choice ${ \cos}^{-1}\left(1-5/\widehat{N}\right)<{\kappa}_0<{ \cos}^{-1}\left(1-10/\widehat{N}\right),$where $\widehat{N}$denotes the mean number of measured gradient directions per shell (Becker et al. 2014). Larger values of *κ*_0_, are appropriate in case of small SNR or large number *N*_*g*_ of gradient directions. For a graphical visualization of this choice and the specific values for the datasets used in this paper, see Fig. 2.

*λ* is the adaptation bandwidth of the procedure that steers the amount of adaptation of msPOAS. For *λ* = 0 the adaptive weights in Eq. (4) vanish for all *m* ≠ *n*. As a consequence, all estimates at any iteration step will coincide with the original data. In contrast, for *λ* = ∞ the adaptive weights coincide with the non-adaptive weights, i.e. ${\tilde{w}}_{mn}^{(k)}={\overline{w}}_{mn}$ for all *m*, *n*, see Eq. (4), and msPOAS finally generates a non-adaptive kernel estimate in the space ${\mathbb{R}}^3\times {\mathbb{S}}^2$ with kernel *K*_loc_, bandwidth ${h}_{k^{\star }}$ and ${\kappa}_{k^{\star }}={\kappa}_0/{h}_{k^{\star }}$.

Basically, *λ* can be chosen to satisfy a *propagation condition* using simulation independent from the processed data (Becker et al. 2012) but for the specific noise distribution. In case of dMRI data, the distribution can be assumed to be a non-central *χ*-distribution with 2*L* degrees of freedom and non-centrality parameter *θ*. We suggest a value of *λ* = 12 for all datasets. Depending on the quality of the estimates for the data-dependent parameters *σ*, *L* slight adjustments may be required, as discussed below. The propagation condition ensures with high probability that in homogenous image regions the msPOAS estimator basically coincides with the non-adaptive in Eq. (1).

### How to Estimate *σ* and *L*?

MsPOAS requires two data-dependent input parameters *σ* and *L*, that fix the properties of the noise distribution and enter the definition of the statistical penalty *s*_*mn*_^(*k*)^, see Eq. (6).

A suitable estimate for the standard deviation *σ* of the noise can be obtained by any method available to the user, see (Aja-Fernández et al. 2009) for a review. The methods typically determine *σ* from the Rayleigh *L* = 1 or central *χ*-distribution *L* > 1 in the image background.

We implemented the method described in (Constantinides et al. 1997) in the toolbox. It can be accessed in the batch editor via SPM -> ACID Toolbox -> Pre-Processing -> Choose POAS options -> Sigma estimation and requires as input the diffusion weighted images from the data, a binary mask file defining background voxel only and the specification of *L*, see below. Running the estimation returns a mean value over all diffusion weighted images. It also writes a **.txt**-file into the data directory with individual values for each diffusion weighted volume. *b*_0_-image typically lead to larger estimates for *σ*, than the diffusion weighted volumes. Note, that the method of Constantinides et al. (1997) does not account for noise correlation due to multi-channel receiver coils (Hutton et al. 2012). Furthermore, non-background structure like ghosts within the defined background mask also leads to overestimates of *σ*. For msPOAS it is advisable to use a conservative small estimate for *σ* and potentially correct a misspecification by adjusting *λ*.

The parameter *L* depends on the reconstruction algorithm, see, e.g., Aja-Fernández et al. (2011). It is very difficult to estimate from the data. For some reconstruction methods it can be shown that *L* = 1 (Sotiropoulos et al. 2013). Generally *L* equals the total number of receiver coils for a Sum-of-Squares reconstruction and is spatially varying for GRAPPA (Aja-Fernández et al. 2011). Fortunately, msPOAS has been shown to be relatively robust against misspecification of *L* (Becker et al. 2014). We therefore suggest to use a value of *L* = 1 consistently in the estimation of *σ* and for msPOAS, if no other estimate is available.

### Experimental Data

Two healthy volunteers (male) participated in the study approved by the local ethics committee after giving written informed consent. The example data used in this paper has been acquired as follows: Experiments were performed on a MAGNETOM Trio, a Tim System 3T scanner (Siemens AG, Healthcare Sector, Erlangen, Germany). Two high-resolution diffusion magnetic resonance imaging (dMRI) data sets were acquired using a reduced field-of-view (FoV) technique (Heidemann et al. 2010), one multi-shell, and one single-shell data set.

For the multi-shell data, the 161 × 58mm FoV was centered on the motor cortex. It had 1.2mm isotropic resolution, and 10 % slice gap, resulting in an effective slice thickness of 1.3mm. The images were acquired at 3 different *b*-values: 21 at *b* = 20s/mm^2^, 100 at *b* = 800s/mm^2^, and 100 at *b* = 2000s/mm^2^ using the directions suggested by Caruyer et al. (2011). The total scan time was about 22 min. This dataset was also used in Becker et al. (2014).

The 156 × 56mm FoV of the single-shell data set was centered on the thalamus with 1mm isotropic resolution, and 10 % slice gap, resulting in an effective slice thickness of 1.1mm. The images were acquired at 2 different *b*-values: 5 at *b* = 0s/mm^2^ and 64 at *b* = 1000s/mm^2^ using the directions provided by Siemens. This dataset was acquired three times, giving a total scan time of about 20 min.

Prior to POAS the data were corrected for motion and eddy current artifacts using the method detailed in Mohammadi et al. (2010), which is implemented as part of the ACID toolbox pipeline. For the analysis in this paper we then estimated the diffusion tensor and FA.

### Parameter Choices for the Multi-Shell Dataset

We repeatedly defined an arbitrary region within the background of the data and used the method implemented in the toolbox and described in the “Methods” section to estimate the noise standard deviation *σ*. We consistently found a value of *σ* = 30. We used *L* = 1 for all calculations as in (Becker et al. 2014).

We used msPOAS parameter values *κ*_0_ = 0.3 and *λ* = 12 to match the choice in Becker et al. (2014). The number of iteration steps was fixed at *k* ^⋆^ = 12 that provided a good balance between computational costs and achieved noise reduction.

### Parameter Choices for the Single-Shell Dataset

Estimation of the noise level in the image background for the single-shell dataset consistently provided a value of *σ* = 45. We used *L* = 1, *k* ^⋆^ = 12, and *λ* = 12 for all calculations. This dataset was measured with three repetitions. First, we smoothed the data for a one-repetition dataset (using the first repetition), here the *κ*_0_ value was used to be *κ*_0_ = 0.5. Then, we smoothed the data for all three repetitions, with *κ*_0_ = 0.3.

Finally, to demonstrate the dependence of the msPOAS outcome on the different method parameters, we consecutively changed one of the three parameters *κ*_0_, *λ*, and *k* ^⋆^, while leaving the others constant. To this end, we used the one-repetition dataset and varied *κ*_0_ = 0.3, 0.5, 0.8, *λ* =1, 5, 10, 12, 50, 100, 500, ∞, *k* ^⋆^ = 4, 8, 12, 16, 20, 24, 28.

### Hardware

We performed the example analysis on a HP Workstation **XW4600** with Intel^®^ Core™2 Duo CPU E6850@3.00GHz and 8GB RAM running with OpenSuSE 12.3 and Matlab 2012b with SPM8.

## Results

The calculations on the described hardware using the optimal parameters given above took approximately 1200 sec = 20 min for the multi-shell dataset, 700 sec ≈ 12 min for the one-repetition single-shell dataset, and 1900 sec ≈ 30 min for the three repetition single-shell dataset.

**Fig. 3:**
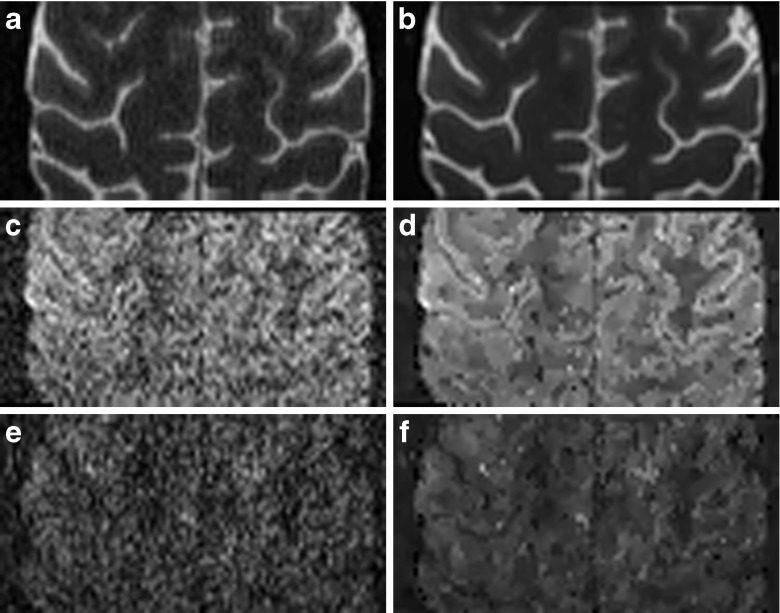
One slice of a diffusion weighted image w/o msPOAS for multi-shell data at all measured *b*-values. (**a**) Original data *b*_0_ -image. (**b**) msPOAS result. (**c**) Original data *b* = 800s/mm^2^. (**d**) msPOAS result. (**e**) Original data *b* = 2000s/mm^2^. (**f**) msPOAS result

In Fig. 3 we compare an axial slice of the multi-shell data for the three used *b*-values 0, 800, 2000s/mm^2^ before and after application of msPOAS. The gray scale has been adjusted such that the *b*_0_-image uses the full range of the scale. For the diffusion weighted images the scale is comparable among them but augmented in comparison with the *b*_0_-image for better contrast.

**Fig. 4:**
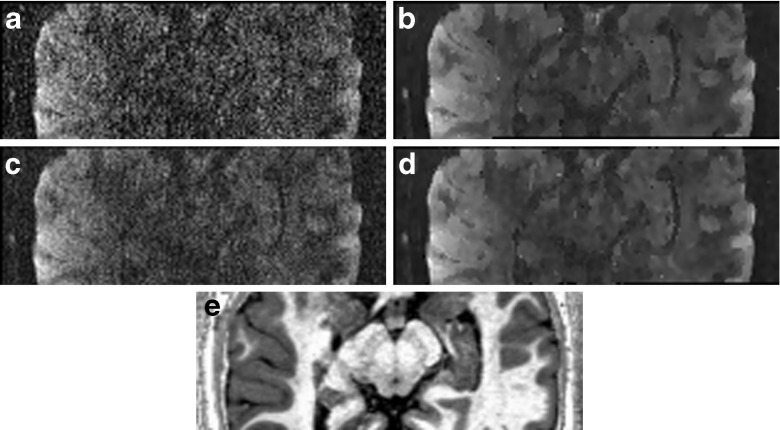
One slice of a diffusion weighted image w/o msPOAS for the one repetition single-shell data (**a** and **b**), compared with the mean of the three-repetition data (**c** and **d**). For the same slice a high-resolution quantitative R1-image was depicted in (**e**) for anatomical reference. The R1 image was acquired with a multi-parameter protocol (Dick et al. 2012; Lutti et al. 2014; Sereno et al. 2013)

In Fig. 4 we show one (randomly selected) diffusion weighted image before and after applying msPOAS, see Fig. 4a and b. For comparison we show an average of all three repetitions of the measurement in Fig. 4c along with the smoothed version in Fig. 4d. The quality of the msPOAS result on the one repetition data outperformed the average of the three repetitions. In Fig. 4e an R1-image of the same slice is shown.

**Fig. 5:**
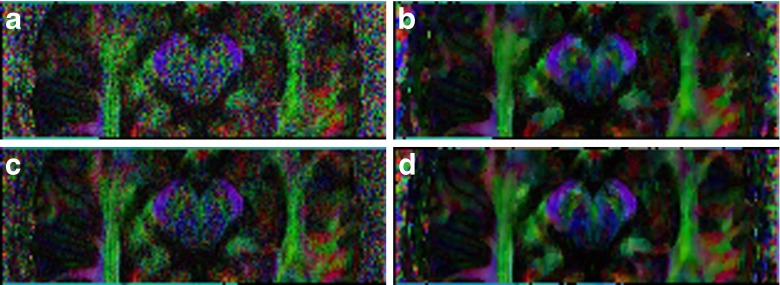
Color coded FA maps for the single shell data w/o msPOAS. (**a**) Original one repetition data. (**b**) msPOAS result on the one repetition data. (**c**) Mean of the three repetition data. (**d**) msPOAS on the mean data

In Fig. 5 we show the corresponding results evaluated in the diffusion tensor model by means of color-coded FA maps.

**Fig. 6:**
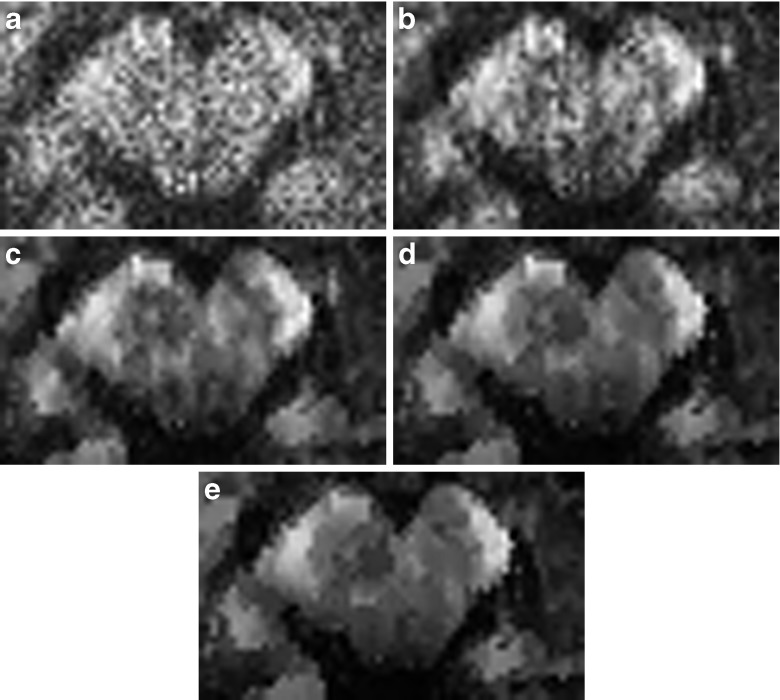
Dependence of FA after msPOAS on *k* ^⋆^ for the one repetition single-shell data. (**a**) *k* ^⋆^ = 0 (Original data). (**b**) *k* ^⋆^ = 4. (**c**) *k* ^⋆^ = 12. (**d**) *k* ^⋆^ = 20. (**e**) *k* ^⋆^ = 28

**Fig. 7:**
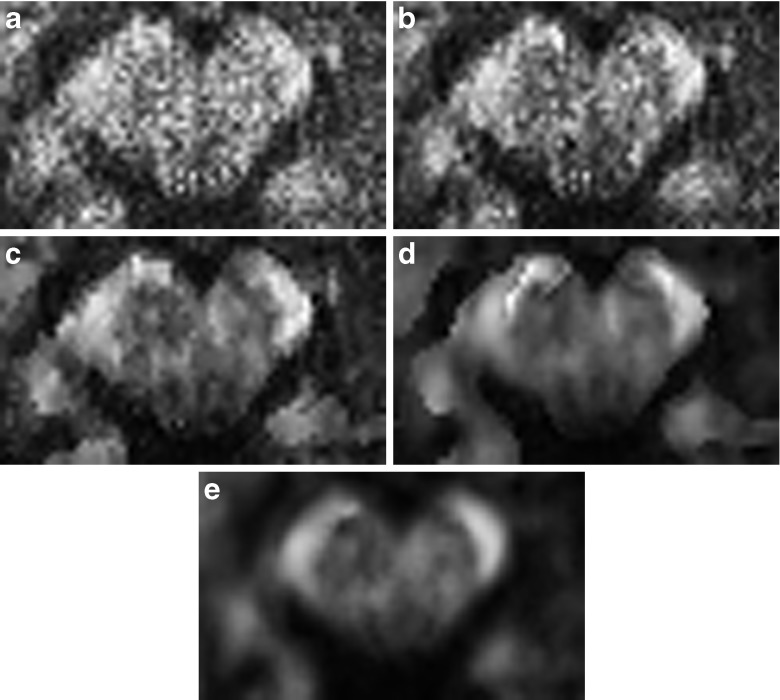
Dependence of FA after msPOAS on *λ* for the one repetition single-shell data. (**a**) λ = 1, (**b**) λ = 5. (**c**) λ = 12. (**d**) λ = 100. (**e**) λ = ∞

**Fig. 8:**
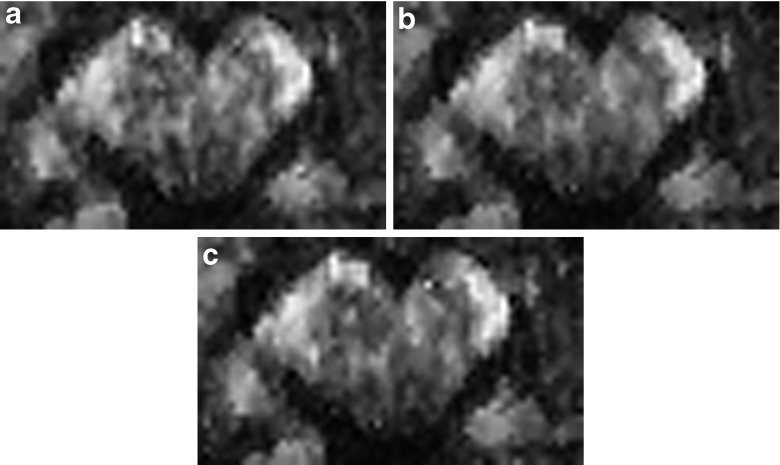
Dependence of FA after msPOAS on *κ*_0_ for the one repetition single-shell data. (**a**) *κ*_0_ = 0.3, (**b**) *κ*_0_ = 0.5, (**c**) *κ*_0_ = 0.8

In Figs. 6, 7, and 8 we show the dependence of the resulting color-coded FA map on the number of iteration steps *k* ^⋆^ (*k* ^⋆^ = 4, 12, 20, 28 only), the adaptation bandwidth *λ* (*λ* = 1, 5, 12, 100, ∞ only), and *κ*_0_, respectively. The amount of noise reduction increases with the number of iteration steps. In homogeneous regions the propagation condition ensures a non-adaptive behavior of msPOAS. Then, with *k* ^⋆^ the achieved variance reduction is increasing. If a region is small compared to the final bandwidth ${h}_{k^{\star }}$ the achievable variance reduction is proportional to the number of voxel in the region, see Eq. (3). *λ* controls the amount of adaptation from full adaptation for very small *λ*, i.e. no smoothing at all, to non-adaptive smoothing for very large values. *κ*_0_ has an influence on the achieved noise reduction via construction, smaller values lead to less noise reduction.

## Discussion

We implemented a method for adaptive denoising diffusion weighted MRI called msPOAS as a toolbox for SPM. The program is part of a general toolbox for artefact correction in diffusion MRI data named ACID.

### Dependence of the Results on the Method Parameters and its Interaction

MsPOAS requires the specification of data-dependent quantities *σ* and *L* and method parameters *h* ^⋆^, *λ*, and *κ*_0_. In this paper, we analyzed the dependence of the outcome of msPOAS on the choice of the method parameters.

As shown in Fig. 6 the effect of noise reduction increases with the number of iteration steps *k* ^⋆^. In homogeneous compartments of the image, the smoothness increases with *k* ^⋆^. On the other hand, the adaptivity of the procedure obviously avoids blurring at the borders. Thus in principle increasing *k* ^⋆^ improves the noise reduction effect of msPOAS without blurring structural border. This can be interpreted as an intrinsic local stopping criterion for the method. However, the computational cost increases exponentially with *k* ^⋆^, see Fig. 1. For very large *k* ^⋆^ deviations in the diffusion weighted images from the structural assumption of local constant image intensities, e.g., by smooth and gradual changes, lead to a step-function approximation of the image intensities, see Becker and Mathé (2013), Becker et al. (2012). Best results are achieved for intermediate *k* ^⋆^, such that a suitable compromise between computation time, variance-controlled bias and required noise reduction has to be made, e.g. *k* ^⋆^ = 12.

The adaptation bandwidth *λ* controls the adaptivity of the method, ranging from complete adaptation at *λ* = 0, where the original is not changed at all by msPOAS, to a non-adaptive estimate at *λ* = ∞, where the adaptivity of msPOAS is turned off, see Fig. 7. Best results can be achieved at *λ* = 12.

Different choices of *λ* have a similar effect on the results of msPOAS as the estimates for *σ*. Generally, if *σ* is underestimated, msPOAS will be to restrictive and only little smoothing effect will appear in the result. If *σ* is overestimated, this may lead to blurring in the result. This also means that the effect a misspecification of *σ* can be compensated (to some extent) by adjusting *λ* accordingly.

The choice of *κ*_0_ influences the amount of smoothing on the sphere. For relatively good SNR *κ*_0_ can be chosen smaller then the recommended value to reduce an estimation bias due to the violation of the a local constant signal function on the sphere. For lower SNR the initial estimates benefit from larger values of *κ*_0_ through stabilization. Our construction of the sequences of bandwidths *h*_*k*_ and *κ*_*k*_, see Eq. (5) and its discussion, automatically lead to increased noise reduction by larger values of *κ*_0_ (with higher computational costs).

The choice of *L* has only a minor effect on the result of msPOAS, see Becker et al. (2014). Nevertheless, msPOAS may benefit from precise specification of *L*, if available. If *L* is unknown, we recommend to use *L* = 1.

### Suggestions for Parameter Choices

We suggest the following procedure for the parameter choices for msPOAS:

1. *L* = 1, if *L* is unknown.
2. Determine *σ* by some suitable method (e.g. the method given in the toolbox) using *L* as previously chosen.
3. *λ* = 12.
4. Choose *κ*_0_ such that $5\le \widehat{N}\left(1- cos\left({\kappa}_0\right)\right)\le 10$, depending on the mean number $\widehat{N}$ of diffusion gradients per shell, see Fig. 2 for a graphical tool for this choice.
5. *k* ^⋆^ = 12.
6. Run msPOAS.
7. Adjust parameters: slightly decrease *λ* if oversmoothing at borders occurs, which looks like Fig. 7d or e. Slightly increase *λ* if the noise reduction in homogeneous regions is less then expected for the utilized *k* ^⋆^: In this case increasing *k* ^⋆^ does not increase noise reduction in homogeneous regions.
8. Adjust *k* ^⋆^ if more or less noise reduction for homogeneous regions is required.
9. Re-run msPOAS if adjustments are necessary.

The evaluation of the msPOAS result can be done at the level of diffusion weighted images or for diffusion model parameters, like FA maps.

## Conclusion

MsPOAS is a powerful method for adaptive noise reduction in diffusion MRI data that is now available as a toolbox for SPM. We demonstrated and discussed the effect of different method parameters and data-dependent quantities on the results of msPOAS and gave recommendations for their choice and determination, respectively.

## Information Sharing Statement

POAS4SPM is part of the ACID-toolbox available at http://www.diffusiontools.com.

SPM is a MATLAB toolbox that is freely available from http://www.fil.ion.ucl.ac.uk/spm/software/spm8/
